# Acceleration of Final Residual Solvent Extraction From Poly(lactide-co-glycolide) Microparticles

**DOI:** 10.1007/s11095-024-03744-9

**Published:** 2024-08-15

**Authors:** Florian Kias, Roland Bodmeier

**Affiliations:** College of Pharmacy, Freie Universität Berlin, Kelchstr. 31, 12169 Berlin, Germany

**Keywords:** microparticles, plasticization, poly(lactide-co-glycolide), solvent extraction, solvent residuals

## Abstract

**Purpose:**

The removal of the residual solvent dichloromethane from biodegradable poly(D,L-lactic-co-glycolic acid) (PLGA) microparticles was investigated by aqueous or alcoholic wet extraction or vacuum-drying.

**Methods:**

Microparticles were prepared by the O/W solvent extraction/evaporation method. The solidified microparticles were separated by filtration and the effect of subsequent drying and wet extraction methods were investigated. The residual solvent content was analysed with gas chromatography (organic solvents) and Karl Fischer titration (water). The effect of extraction conditions on microparticle aggregation, surface morphology and encapsulation of the drugs dexamethasone and risperidone was investigated.

**Results:**

Residual dichloromethane was reduced to 2.43% (w/w) (20 °C) or 0.03% (w/w) (35 °C) by aqueous wet extraction. With vacuum-drying, residual dichloromethane only decreased from about 5% (w/w) to 4.34% (w/w) (20 °C) or 3.20% (w/w) (35 °C) due to the lack of the plasticizing effect of water. Redispersion of filtered, wet microparticles in alcoholic media significantly improved the extraction due to an increased PLGA plasticization. The potential of different extractants was explained with the Gordon-Taylor equation and Hansen solubility parameters. Extraction in methanol: or ethanol:water mixtures reduced residual dichloromethane from 4 - 7% (w/w) to 0.5 - 2.3% (w/w) within 1 h and 0.08 - 0.18% (w/w) within 6 h. Higher alcohol contents and higher temperature resulted in aggregation of microparticles and lower drug loadings.

**Conclusion:**

The final removal of residual dichloromethane was more efficient with alcoholic wet extraction followed by aqueous wet extraction at elevated temperature and vacuum drying of the microparticles.

## Introduction

Common manufacturing techniques for biodegradable microparticles such as solvent extraction/evaporation, organic phase separation or spray drying require the initial dissolution of PLGA in organic solvents such as dichloromethane (DCM), chloroform, or ethyl acetate [1, 2]. Due to their toxicity and effect on microparticle properties and stability, the solvents must be removed during the manufacturing process [3, 4]. Extraction must ensure that the permitted daily exposure with solvents recommended by the ICH (e.g. 6 mg/d DCM) is not exceeded when patients are treated with the final product [5]. Diafiltration or dilution can be used to control the solvent removal in the solvent extraction/evaporation process and thus modify the properties of the final microparticles, such as size, morphology, drug loading and release [4, 6, 7]. Diafiltration alone did not affect residual solvent content significantly, but an additional increase in temperature did. The final extraction essentially only depends on the diffusivity of the organic solvent in the dispersed phase, which decreases with decreasing organic solvent content und thus increasing viscosity [8, 9]. Particularly after solidification, when the glass transition temperature falls below process temperature and thus the polymer turns into the glassy state, the extraction slows down significantly [8–10]. Diffusion in a rubbery or glassy polymer results from elementary jumps of molecule segments, so-called jumping units, between vacancies in the polymer, so-called hole free volume, which decreases with decreasing plasticization by the residual solvent content [11–13]. Meeting the regulatory specification for residual solvent content may require long process times and secondary drying techniques, utilizing for example elevated temperature, decreased pressure, lyophilization and supercritical carbon dioxide extraction [14–18]. To reduce the residual solvent content already before separation of the microparticles various non-solvents for PLGA can be added to the continuous phase. The use of organic non-solvents for extraction is described primarily for the phase separation technique (e.g. silicon oils, alkanes [14, 19]), but also for the solvent extraction/evaporation technique (e.g. methanol [20], ethanol [21–23], isopropanol [24]). However, in most cases there is no information about the effect of the extractants and their concentration on the removal rate of residual solvents. The addition of alcohols (especially ethanol) to the continuous aqueous phase or post-processing with alcoholic media has been studied in more detail as modifier for surface porosity and thus drug release. Ethanol can reduce the initial burst release of drugs [25] and prolong [26] or eliminate a potential lag phase [22]. Particularly elevated temperatures and long process times of ethanolic treatment may reduce encapsulation efficiency by leaching out the drug and favour degradation of PLGA. The plasticizing effect of ethanol is crucial for almost all the described consequences, in particular the pore closure, the degradation of PLGA and the improved extraction of residual solvents. Absorbed non-solvents are stored between the polymer chains, reducing the glass transition temperature and increasing the free volume and diffusivity of all components within the matrix [27, 28]. The plasticizing effect of the alcohols facilitate the rearrangement of the PLGA chains even in the absence of dichloromethane, thus potentially preventing the subsequent polymer “ageing” and reducing the porosity which might be caused by secondary drying methods like lyophilization [16, 18, 29]. The depression of glass-transition temperature with increasing solvent content can be calculated, using the Gordon-Taylor equation (Eq. 1). For this, the weight fractions 

${{\varvec{w}}}_{\mathbf{P}}$ and ${{\varvec{w}}}_{\mathbf{S}}$ and the glass transition temperatures ${{\varvec{T}}}_{{\varvec{g}}{\varvec{P}}}$ and ${{\varvec{T}}}_{{\varvec{g}}{\varvec{S}}}$ of the polymer **P** and the solvent **S** are used. The constant ${\varvec{k}}$ can be estimated using the corresponding densities ${{\varvec{p}}}_{\mathbf{P}}$ and ${{\varvec{p}}}_{\mathbf{S}}$ and the glass transition temperatures (Eq. 2) [30].

1 $${{{\varvec{T}}}_{{\varvec{g}}}}_{{\varvec{S}}{\varvec{y}}{\varvec{s}}{\varvec{t}}{\varvec{e}}{\varvec{m}}}=\frac{{{\varvec{w}}}_{\mathbf{S}}{{\varvec{T}}}_{{\varvec{g}}{\varvec{S}}}+{\varvec{k}}{\boldsymbol{ }{\varvec{w}}}_{{\varvec{P}}\boldsymbol{ }}{{\varvec{T}}}_{{\varvec{g}}{\varvec{P}}}}{{{\varvec{w}}}_{\mathbf{S}}+\mathbf{k}{{\varvec{w}}}_{\mathbf{P}}}$$

2 $${\varvec{k}}=\frac{{{\varvec{p}}}_{{\varvec{S}}}{{\varvec{T}}}_{{\varvec{g}}{\varvec{S}}}}{{{\varvec{p}}}_{{\varvec{P}}}{{\varvec{T}}}_{{\varvec{g}}{\varvec{P}}}}$$

Water can also act as a plasticizer for PLGA, but its effect on glass-transition temperature is rather small [18, 30]. Furthermore, water may hydrate the individual molecular chains only to a very limited extent during manufacturing and is instead more likely present in pores or cavities, due to its low solubility in dichloromethane and PLGA [10]. Similarly, only the molecularly dissolved portion of encapsulated drug can lower the glass transition temperature of the polymer [31, 32]. The solubility or miscibility of solvents and drugs in PLGA can be estimated using the Hansen solubility parameters (HSP) [33, 34]. The partial solubility parameters, dispersive interactions ${{\varvec{\delta}}}_{{\varvec{d}}}$, polar interactions ${{\varvec{\delta}}}_{{\varvec{p}}}$ and hydrogen bonding ${{\varvec{\delta}}}_{\mathbf{h}}$, are assigned to a substance so that it can be positioned in a three-dimensional coordinate system. The smaller the distance ${{\varvec{R}}}_{{\varvec{a}}}$ of the coordinates between two substances, the higher their principal affinity and thus expected solubility in each other.

3 $${{\varvec{R}}}_{{\varvec{a}}}=\sqrt{4{({{\varvec{\delta}}}_{{\varvec{d}}1}-{{\varvec{\delta}}}_{{\varvec{d}}2})}^{2}+{({{\varvec{\delta}}}_{{\varvec{p}}1}-{{\varvec{\delta}}}_{{\varvec{p}}2})}^{2}+{({{\varvec{\delta}}}_{{\varvec{h}}1}-{{\varvec{\delta}}}_{{\varvec{h}}2})}^{2}}$$

In this study the final removal of dichloromethane from dry and wet solidified PLGA microparticles was investigated. The effect of various alcoholic non-solvents in the continuous phase on the dichloromethane removal, microparticle agglomeration and loss of encapsulated dexamethasone (slightly soluble in dichloromethane and PLGA [35, 36]) and risperidone (freely soluble in dichloromethane and PLGA [35, 37]) was examined. The results were related to the plasticizing effect of the non-solvents and their affinity to PLGA and the encapsulated drugs.

## Materials and Methods

### Materials

Micronized dexamethasone (DEX) (Caesar & Loretz GmbH, Hilden, Germany); risperidone (RIS) (RPG Life Sciences limited, Navi Mumbai, India); poly(lactide-co-glycolide) (PLGA) (Resomer® RG 503H, Evonik Industries AG, Darmstadt, Germany); acetonitrile (HPLC grade), glycerol, isopropanol (IPA) (technical grade), methanol (MeOH) (HPLC grade) (VWR International GmbH, Darmstadt, Germany); dichloromethane (DCM) (HPLC grade), dimethyl sulfoxide (DMSO) (headspace grade), propylene glycol (PG) (Carl Roth GmbH + Co. KG, Karlsruhe, Germany); ethanol absolute (EtOH), polyvinyl alcohol 4–88 (PVA) (Merck KGaA, Darmstadt, Germany).

### Methods

#### Preparation of Microparticles

PLGA microparticles were prepared in the solvent extraction/evaporation process. The organic drug:PLGA:dichloromethane phase was prepared by dispersing or dissolving 0 - 40% (w/w) dexamethasone or risperidone (based on polymer weight) in a 10% (w/w) PLGA in dichloromethane solution for 1 min (VF2, IKA-Werke GmbH & Co. KG, Staufen im Breisgau, Germany). 2.5 mL of the drug:PLGA solution was emulsified into 50 mL 0.25% (w/v) PVA solution as continuous phase using a propeller stirrer (d = 3.5 cm, 800 rpm). To study conventional solvent extraction, parts of the batch were sampled over time and subsequently separated by vacuum filtration using a 10 µm stainless-steel sieve. If drug was encapsulated, the entire batch was transferred into 450 mL of continuous phase 15 min after the start of emulsification and stirred with a magnetic stirrer. To study non-solvent assisted extraction blank microparticles were filtered 2.5 h and drug loaded microparticles 30 min after emulsification. Filtered microparticles were washed three times by pouring 250 mL of deionized water into the suction filter, briefly redispersing the microparticles with a spatula and removing off the water by vacuum. Subsequently they were analysed for solvent content, dried, or redispersed in 50 mL aqueous continuous phase containing 0 - 50% (w/w) non-solvent, to further decrease solvent content. This continuous phase was sampled over time and microparticles were separated, washed, and analysed in the same way as untreated samples. Microparticles were dried in vacuum at 35 °C for determination of encapsulation efficiency and stored in a desiccator at 7 °C.

#### Residual Solvent Content

The water content of microparticles was determined according to the method for coulometric Karl Fischer determination (Ph. Eur. 2.5.32). 10.0 - 20.0 mg microparticles were accurately weighed and dissolved in 1.0 mL acetonitrile. About 250.0 mg of this solution, accurately weighed, were analysed in triplicate for the water content with a coulometric Karl Fischer titrator (831 KF Coulometer, Metrohm AG, Herisau, Switzerland) using HYDRANDAL Coulomat AD (Honeywell Specialty Chemicals Seelze GmbH, Seelze, Germany). The water content based on total weight was calculated after correction for the water content of a blank.

Dichloromethane and alcohol content of microparticles was quantified with headspace gas chromatography (GC-2014, Shimadzu Corp., Kyoto, Japan) with a method adapted from USP monograph for residual solvents using a capillary column equivalent to USP G43 phase (Rtx-1301, Restek Corp., Bellefonte, USA). 10.0 - 50.0 mg microparticles, dissolved in 5.0 mL dimethyl sulfoxide, were sealed in a 20 mL headspace GC vial with an aluminium screw cap with PTFE septum. Samples were equilibrated automatically under shaking by an autosampler (AOC-6000, Shimadzu Corp., Kyoto, Japan) for 45 min at 105 °C. 1.0 mL of the gas phase was sampled automatically, with a needle temperature 5 °C above previous equilibration temperature and injected at 140 °C with a subsequent split ratio of 5. The column oven temperature was maintained at 40 °C and increased after 7 min to 120 °C (DMSO) with a heating rate of 30 K/min. The carrier gas was nitrogen. Samples were detected with a flame ionization detector (FID) set to 250.0 °C. Evaluation of the spectra was performed with LabSolutions 5.98 (Shimadzu Corp., Kyoto, Japan). The dichloromethane and alcohol content in the samples was calculated from peak areas using linear calibration curves obtained by dilution series. For filtered wet microparticles, the water content was deducted from the sample weight to calculate their dichloromethane content.

#### Solubility of Dichloromethane

The solubility of dichloromethane in aqueous mixtures of 0 - 50% (w/w) of various alcoholic non-solvents was investigated. 15 g of non-solvent:water mixture were added into a 20 mL vial, filled up with 3 - 5 mL dichloromethane, sealed and shaken at room temperature for 24 h. The vial was then left for another 24 h to allow the two phases to separate. 0.5 mL was sampled from the aqueous supernatant, diluted, and examined using headspace GC. For this purpose, the previously mentioned method was adapted as follows: 5 mL sample were incubated in a 20 mL GC vial for 60 min at 80 °C. The column oven temperature was maintained at 80 °C and heated to 160 °C after 7 min. The dichloromethane content in the samples was calculated from peak area using a linear calibration curve obtained by dilution series.

#### Optical Microscopy

For microscopic images, samples were observed on a glass slide under polarized light microscope (Axioscope) equipped with an Axiocam 105 colour camera (Carl Zeiss Microscopy GmbH, Jena, Germany) and images were processed by the software Zen 3.2 (Carl Zeiss Microscopy GmbH, Jena, Germany).

#### Drug Loading and Encapsulation Efficiency

The actual drug loading was determined by dissolving 10.0 - 20.0 mg microparticles in acetonitrile and diluting 1:1 (V/V) with deionized water. The absorbance of the solution was then measured by UV-Vis spectroscopy (UV-1900i, Shimadzu Corp., Kyoto, Japan) at 242 nm (dexamethasone) or 276 nm (risperidone). Concentrations were calculated with the use of standard curves. The encapsulation efficiency (%) was calculated as the ratio of actual drug loading to the theoretical drug loading.

#### Surface Morphology

Scanning electron microscopy (SEM) (SU8030, Hitachi High-Technologies Europe GmbH, Krefeld, Germany) was used to image the surface morphology of microparticles. Samples were sputtered under an argon atmosphere with 5 nm gold (CCU-010 HV, Safematic GmbH, Zizers, Switzerland) and then observed.

## Results and Discussion

PLGA microparticles prepared in a solvent extraction/evaporation process solidified at a residual dichloromethane content of 5 - 10% (w/w). Based on the Gordon-Taylor equation (Eq. 1) and literature data (Table I), the glass transition temperature of PLGA 503H would only decrease below ambient temperature at about 3.5% (w/w) residual dichloromethane. However, residual solvents are not necessarily homogeneously distributed within microparticles and an outer skin may solidify completely with higher solvent content towards the center of the microparticles [10].

**Table I:** Density and glass transition temperature of excipients (references in parenthesis) used to calculate glass transition temperature of binary systems with Gordon-Taylor equation

Excipients	$p$ at 25 °C (g/cm^2^)	T_G_ (K)
PLGA 503H	1.580 [30]	319 [30]
DCM	1.326 [40]	100 [26]
Water	0.997 [40]	138 [30]
MeOH	0.786 [40]	103 [41]
EtOH	0.785 [40]	97 [42]
IPA	0.781 [40]	115 [43]
PG	1.033 [40]	167 [44]
Glycerol	1.258 [40]	191 [44]

**Figure 1:**
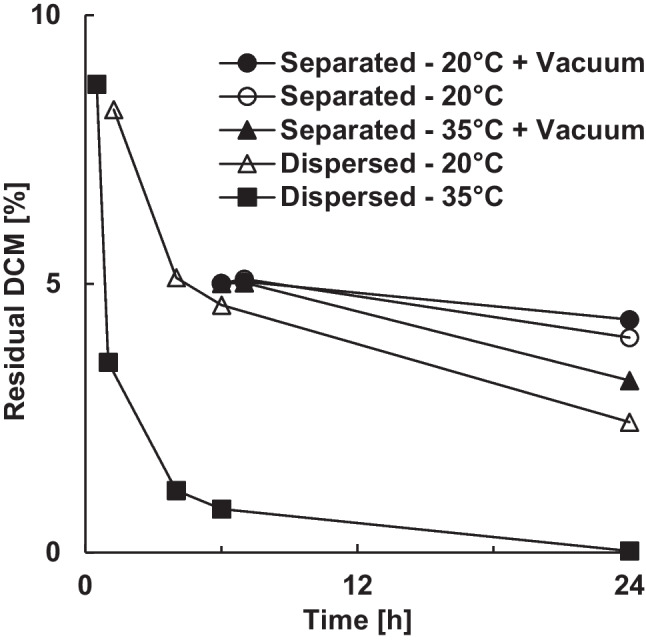
Effect of extraction and drying conditions on the residual dichloromethane content of blank PLGA microparticles.

After 24 h stirring in the continuous phase, the microparticles still contained 2.4% (w/w) residual dichloromethane, due to the slow final extraction (Fig. 1). If the solidified microparticles were filtered after 6 h with a residual dichloromethane content of around 5% (w/w), the dichloromethane removal slowed down even more compared to microparticles dispersed in continuous phase. Water acted as a plasticizer in the wet extraction process and thus improved the diffusion of dichloromethane out of the PLGA matrix [30]. Vacuum drying hardly improved the solvent removal from filtered microparticles, since it was not the evaporation of the highly volatile dichloromethane that limited the final extraction, but rather the low diffusivity within PLGA [8]. A parallel increase of temperature to 35 °C only slightly improved the extraction, so that 3.2% (w/w) dichloromethane remained in the microparticles after a total process time of 24 h. Due to the low glass transition temperature of PLGA, a further increase in temperature was hardly possible without the microparticles sticking together. Increasing the temperature in the wet extraction process, i.e. in combination with the plasticizing effect of water, resulted in a significantly improved extraction. A residual dichloromethane content of 0.8% (w/w) after 6 h and 0.03% (w/w) after 24 h was achieved. However, increasing the temperature may affect the stability of PLGA and some drugs and promote drug leaching [26].

PLGA microparticles with a theoretical risperidone loading of 40% (w/w) had an encapsulation efficiency of 88% (data not shown). Barely any drug leached out, stirring the microparticles at ambient temperature. 85% of risperidone remained encapsulated after 24 h. Increasing the temperature to 35 °C after solidification, decreased encapsulation efficiency to 75% within 4 h. Between 4 and 24 h, the encapsulation efficiency was reduced to 7% due to partial erosion of the microparticles (data not shown). This was caused by hydrolysis of PLGA, enhanced by risperidone, and elevated temperature [26, 38]. Increasing the temperature for solvent extraction should therefore only be used for short time and with caution. A modification of the continuous aqueous phase was investigated as an alternative to improve the extraction.

To evaluate the potential of various non-solvents having at least one hydroxyl group for wet extraction, solidified PLGA microparticles with a residual dichloromethane content of 5 - 6% were filtered, washed, and redispersed in water containing different alcohols and amounts. The residual dichloromethane content of blank microparticles after 6 h was decreased even less by 50% (w/w) Glycerol (3.6% (w/w) DCM) or Propylene glycol (PG) (4.4% (w/w) DCM) compared to pure water (3.2% (w/w) DCM). The addition of the monohydric alcohols methanol (MeOH), ethanol (EtOH) and isopropanol (IPA) reduced the residual dichloromethane content significantly, so these were further investigated. A potential explanation was a lower solubility of dichloromethane in the aqueous continuous phase by addition of polyhydric alcohols, compared to monohydric alcohols (Table II).

**Table II:** Effect of the type and amount of alcohol in the continuous phase on the dichloromethane solubility [mg/mL]

Alcohol content [% (w/w)]	0	20	50
MeOH	11	19	73
EtOH	11	14	147
IPA	11	11	27
PG	11	14	20
Glycerol	11	8	13

The diffusivity in the PLGA matrix was ranked as more important for the final extraction from solidified microparticles than the solution capacity in the continuous phase [10]. The HSP distance R_a_ (Eq. 3) between the monohydric alcohols and PLGA is smaller compared to polyhydric alcohols and PLGA, showing the higher affinity and solubility of the first, although their distance appears still large compared to dichloromethane and PLGA (Table III).

**Table III:** Hansen solubility parameters of the investigated drugs and excipients (references in parenthesis) and their HSP distance R_a_ to PLGA

Excipients	${\delta }_{d}$	${\delta }_{p}$	${\delta }_{h}$	Method	R_a_ PLGA
PLGA 50:50	16.4	8.7	3.6	Solubility testing by turbidity [28]	-
DEX	20.5	15.4	8	Group contribution by Hoftyzer and van Krevelen [42]	11.5
RIS	18.5	8.4	8.1	Molecular dynamic simulation [37]	6.2
DCM	17	7.3	7.1	Group contribution by Barton [40]	4.0
Water	15.5	16	42.3	Group contribution by Barton [40]	39.4
MeOH	14.7	12.3	22.3	Group contribution by Barton [40]	19.3
EtOH	15.8	8.8	19.4	Group contribution by Barton [40]	15.8
IPA	15.8	6.1	16.4	Group contribution by Barton [40]	13.1
PG	16.8	9.4	23.3	Group contribution by Barton [40]	19.7
Glycerol	9.3	15.4	31.4	Group contribution by Barton [40]	31.9

**Figure 2:**
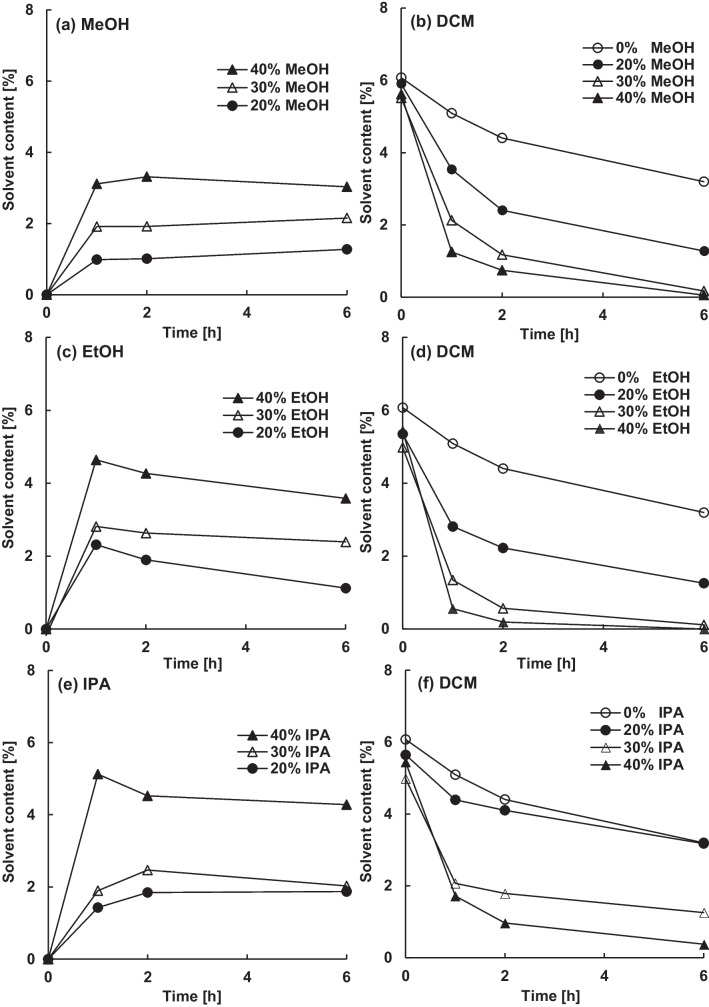
Effect of the amount of methanol (**a**, **b**), ethanol (**c**, **d**) or isopropanol (**e**, **f**) in the continuous phase on the content of absorbed alcohol (**a**, **c**, **e**) and residual dichloromethane (**b**, **d**, **f**) in blank microparticles.

Because of the increased affinity to PLGA and their smaller molecule size, monohydric alcohols diffused better into the matrix, where they increased the free volume and lowered the glass transition temperature. Although the removal of absorbed alcohols was not examined in this study, their permissible limits in the final product, 30 mg/d methanol, 50 mg/d ethanol and 50 mg/d isopropanol, are significantly higher than for dichloromethane (6 mg/d), due to their lower toxicity [5]. In general, increasing the alcohol concentration in the continuous phase, increased the content of absorbed alcohol in the microparticles (Fig. 2).

As the chain length of the monohydric alcohols increased, the HSP distance to PLGA decreased. Accordingly, the order of alcohol absorption was isopropanol > ethanol > methanol, despite a decrease in molecular size (4.3% (w/w) isopropanol, 3.6% (w/w) ethanol or 3.0% (w/w) methanol after 6 h in 40% (w/w) alcohol). However, the residual dichloromethane content did not correspond to this trend and was lowest in ethanol, followed by methanol and isopropanol (Fig. 2).

**Figure 3:**
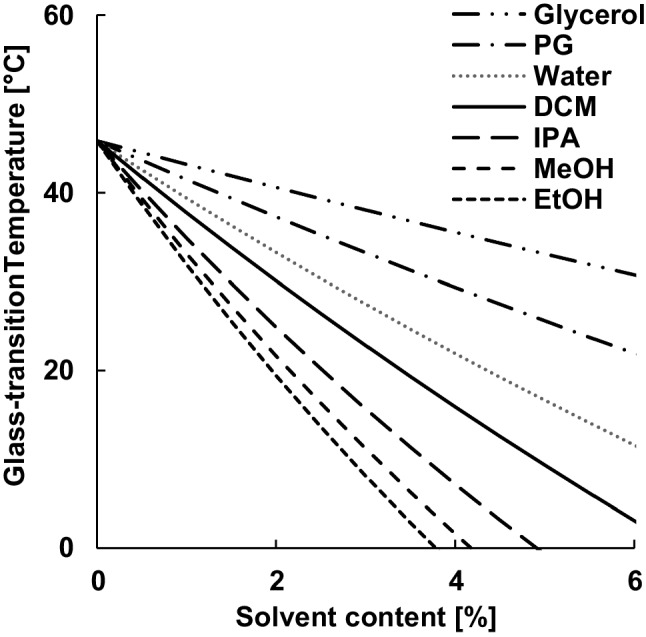
Effect of the type and amount of absorbed (non-)solvent on the glass-transition temperature of PLGA, calculated with the Gordon-Taylor equation.

Not only the content of absorbed non-solvent but also its plasticizing potency was crucial for the extraction of residual dichloromethane. The effect of (non-)solvents on the glass transition temperature of the polymer was estimated using the Gordon-Taylor equation (Eq. 1). According to the density and the glass transition temperature of the different excipients (Table I), the plasticizing effect was in order of ethanol > methanol > isopropanol > dichloromethane (Fig. 3).

**Figure 4:**
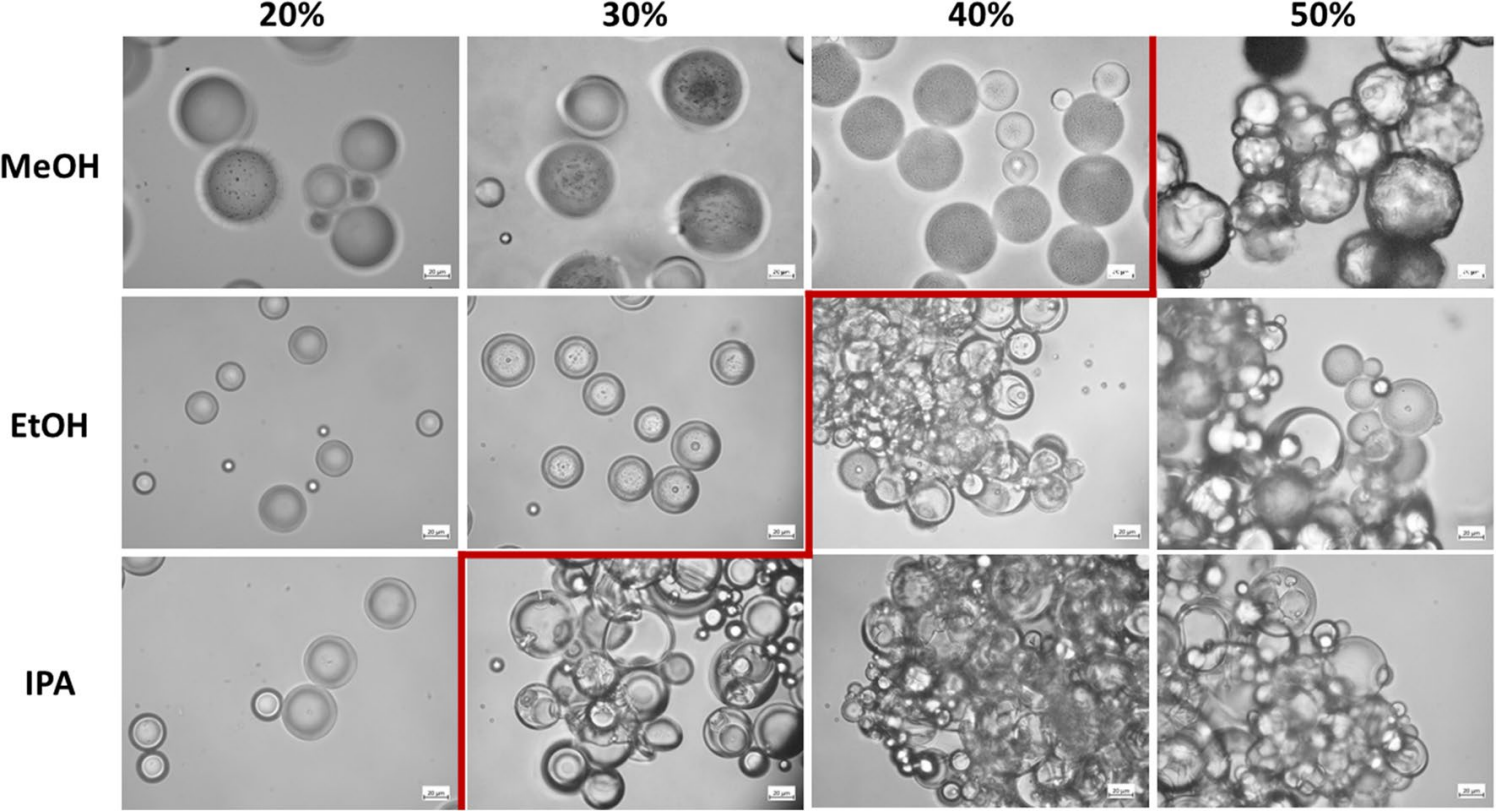
Effect of the type and amount of alcohol in the continuous phase on the microscopic appearance of blank microparticles after 6h (the red line marks the limit when sticking occurred).

Microparticles that were redispersed in up to 50% (w/w) propylene glycol or glycerol did not agglomerate and had no optical changes compared to microparticles redispersed in water. With increasing molecular weight of monohydric alcohols, the microparticles agglomerated and stuck together at lower content of absorbed alcohol (Fig. 4). The dispersibility of the microparticles was only retained up to 40% (w/w) methanol, 30% (w/w) ethanol or 20% (w/w) isopropanol. This trend contradicted the calculated plasticizing effects, according to which ethanol reduced the glass transition temperature of PLGA the most and was therefore the most likely to make it sticky. Rather, the trend of HSP distances was reflected here (Table III). Although isopropanol is a poor solvent for PLGA, it had the shortest HSP distance among the alcohols examined. As it was perhaps the most likely to solve the surface of the microparticles, it made them sticky at the lowest concentration. Since isopropanol addition caused instabilities and had the least effect on the glass transition temperature and residual dichloromethane content, further work focused on methanol and ethanol. Alcohol-assisted solvent extraction could potentially be accelerated by elevating the temperature. However, this may cause drug loss and degradation of PLGA and drug. In addition, the flash point of the water:alcohol mixture increases with increasing alcohol content and is 20 - 35 °C at the concentrations used, which should be taken into account for safety reasons [39].

**Figure 5:**
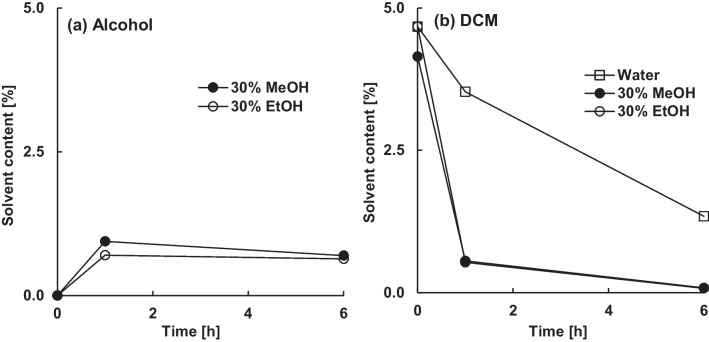
Effect of the type and amount of alcohol in the continuous phase on the content of absorbed alcohol (**a**) and residual dichloromethane (**b**) in the microparticles (30% theoretical dexamethasone loading).

Microparticles loaded with dexamethasone absorbed less methanol or ethanol than blank microparticles (Fig. 5 a). They only contained 0.6% (w/w) or 0.7% (w/w) instead of 2.4% (w/w) or 2.2% (w/w) of methanol or ethanol after 6 h in 30% (w/w) non-solvent. Despite the lower content of absorbed alcohol, dichloromethane was removed efficiently. A residual dichloromethane content below 0.1% (w/w) was achieved with both alcohols within 6 h (Fig. 5 b).

Due to its low solubility in PLGA [35], dexamethasone was present almost entirely as dispersed crystals in the microparticles. These absorbed neither dichloromethane nor alcohols, but contributed to the mass of the microparticles and thereby reduced the calculated solvent contents.

**Figure 6:**
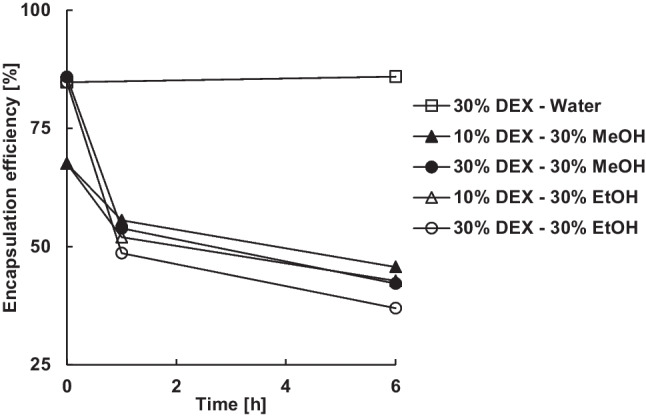
Effect of the theoretical dexamethasone loading and type and amount of alcohol in the continuous phase on the encapsulation efficiency.

The encapsulation efficiency of dexamethasone was significantly reduced by methanol and ethanol (Fig. 6). While the encapsulation efficiency of microparticles with a theoretical drug loading of 30% (w/w) remained unchanged at around 85% during extraction in water, it decreased to 42% in methanol and 37% in ethanol after 6 h.

**Figure 7:**
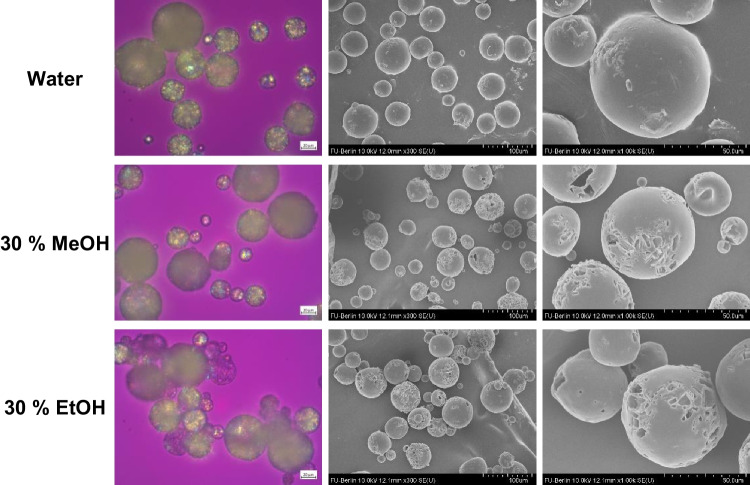
Effect of type and amount of alcohol in the continuous phase on the appearance of microparticles (30% theoretical dexamethasone loading) after 6 h extraction under polarized light microscope (left) and scanning electron microscope (mid and right).

As a result of extraction, some microparticles apperently no longer contained crystalline drug on the surface or even none at all (Fig. 7). Both alcohols increased the solubility of dexamethasone in the continuous phase as well as in PLGA, increasing the concentration gradient and thus the diffusion out of the microparticles. In addition, particles with a high loading of dispersed drug tend to percolate during in-vitro release, which means that drug crystals close to the surface dissolve, creating pores that allow access to further drug crystals. The result is a pore network that enables the rapid initial burst release of drug. Extraction with alcohol also left pores on the surface of microparticles with 30% loading in the rectangular shape and size of dexamethasone crystals indicating percolation (Fig. 7). However, also microparticles with only 10% theoretical dexamethasone loading, i.e. presumably far below the percolation threshold, showed a reduction in encapsulation efficiency from 68 to 45% or 43% after 6 h in 30% methanol or ethanol.

**Figure 8:**
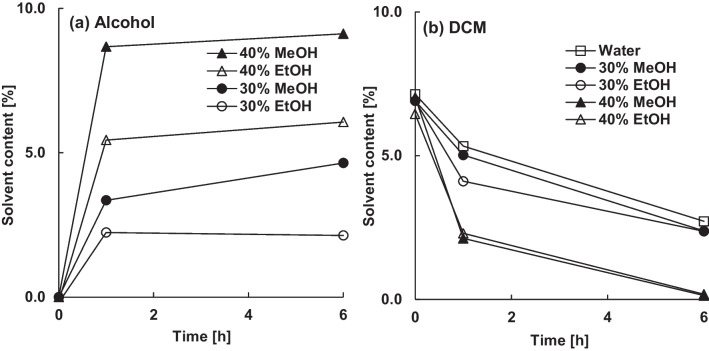
Effect of of the type and amount of alcohol in the continuous phase on the content of absorbed alcohol (**a**) and residual dichloromethane (**b**) in the microparticles (30%  theoretical risperidone loading).

Encapsulated risperidone resulted in increased absorption of alcohols, especially methanol, compared to blank microparticles (Fig. 8 a). After 6 h in 30% (w/w) methanol the microparticles contained 4.6% (w/w) alcohol. Despite the increased alcohol absorption, dichloromethane was removed less efficient compared to blank or dexamethasone loaded microparticles (Fig. 8 b). 30% (w/w) ethanol in particular accelerated dichloromethane extraction within the first hour due to its potent plasticizing effect. However, the residual dichloromethane content of 2.4% (w/w) after 6 h in 30% (w/w) methanol or ethanol was comparable to 2.7% (w/w) in water.

**Figure 9:**
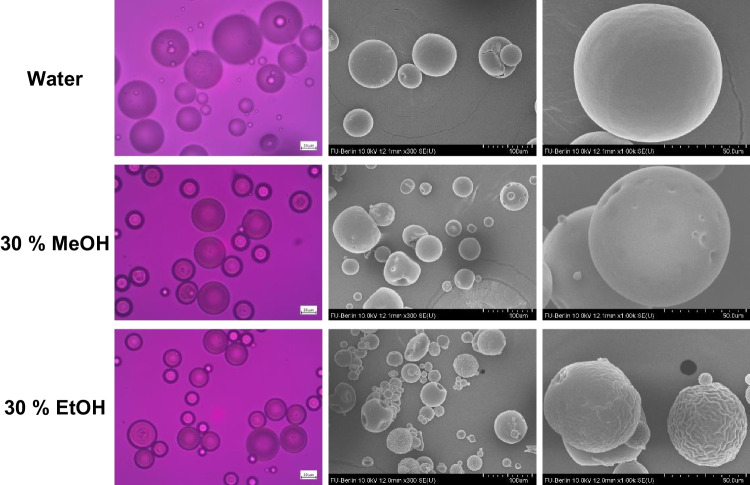
Effect of type and amount of alcohol in the continuous phase on the appearance of microparticles (30% theoretical risperidone loading) after 6h extraction under polarized light microscope (left) and scanning electron microscope (mid and right).

The plasticizing effect of both alcohols was evident from changes of particle surface (Fig. 9). Extraction in both alcohols caused small dents, possibly due to the polymer skin collapsing into underlying cavities. Additionally, ethanol resulted in a wrinkled surface, due to its greater plasticization effect. In contrast to dexamethasone, risperidone was mainly dissolved in PLGA matrix, as observed under polarized light microscope. Only the molecularly dissolved fraction of encapsulated drug affected the mobility of polymeric chains and therefore the glass transition temperature, solubility and diffusivity [31, 32]. Thus, risperidone was able to increase the affinity of dichloromethane and alcohols to PLGA.

**Figure 10:**
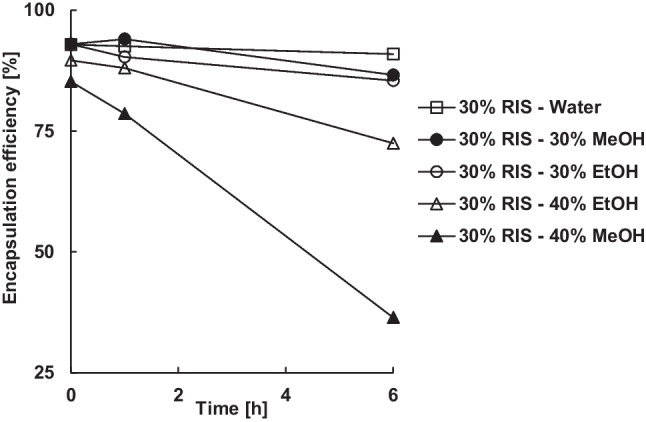
Effect of the type and amount alcohol in the continuous phase on the encapsulation efficiency of 30% risperidone.

Risperidone was barely extracted in 30% (w/w) alcohol (Fig. 10), although it was dissolved and therefore diffusible, due to its significantly higher affinity for PLGA (R_a_ = 6.2) and residual dichloromethane (R_a_ = 3.3) compared to methanol (R_a_ = 16.6) and ethanol (R_a_ = 12.5). Within 6 h, the encapsulation efficiency decreased only slightly from 92 to 87% or 85% in 30% (w/w) methanol or ethanol. Increasing the content of methanol or ethanol in the continuous phase to 40% (w/w) reduced the residual dichloromethane content to below 0.2% (w/w) after 6 h (Fig. 8 b). However, the encapsulation efficiency was also reduced significantly to 36% by methanol or 72% by ethanol and the microparticles stuck together due to the strong plasticizing effect.

## Conclusions

The removal of residual dichloromethane from PLGA microparticles was more efficient with alcoholic wet extraction, followed by aqueous wet extraction at elevated temperature and vacuum drying of the microparticles. The effect of the type and the amount of various alcohols on the residual solvent content, microparticle agglomeration and drug loss was investigated in detail, resulting in a solvent extraction process that reduces time for solvent removal, eliminate the need for energy-intensive secondary drying and thus improving efficiency of microparticle manufacturing. The potential and limitations of different extractants was explained by the Gordon-Taylor equation and Hansen solubility parameters. This approach can be used to evaluate the potential of alcoholic and other extractants in dependence of polymer and drug properties. Further research is necessary to reduce loss of encapsulated drug and thus utilize the full potential of non-solvent-assisted extraction of residual solvents from microparticles.

## Data Availability

The datasets generated during and/or analysed during the current study are available from the corresponding author on reasonable request.
